# Cost of illness for colorectal cancer in Japan - a time trend and future projections (1996–2035) based on governmental statistics

**DOI:** 10.1186/s12913-023-09831-8

**Published:** 2023-08-22

**Authors:** Kunichika Matsumoto, Yosuke Hatakeyama, Kanako Seto, Ryo Onishi, Koki Hirata, Yinghui Wu, Tomonori Hasegawa

**Affiliations:** 1 Department of Social Medicine, Toho University School of Medicine, 5-21-16 Omori-nishi, Ota-ku, Tokyo, 143-8540 Japan; 2 School of Nursing, Shanghai Jiao Tong University, 227 South Chongqing Road, Shanghai, China

**Keywords:** Cost of illness, Colorectal cancer, Japan, Health economics, Health policy

## Abstract

**Background:**

In Japan, the crude mortality rate of colorectal cancer is the second highest among men and highest among women by site. We aimed to calculate the social burden of colorectal cancer using the cost of illness (COI) method and identify the main factors that drove changes in the COI.

**Methods:**

From 1996 to 2020, the COI was estimated by summing direct, morbidity, and mortality costs. In addition, the COI by 2035 was projected by fitting approximate curves obtained from historical data to health-related indicators by sex and age. Future projections of the number of patients by the stage of disease were also made to explore the factors that changed the COI.

**Results:**

The number of deaths and incidence from colorectal cancer was expected to continue increasing due to population aging. However, the COI was projected to rise from 850.3 billion yen in 1996 to 1.451 trillion yen in 2020, and peaked at 1.478 trillion yen in 2023 before it declined.

**Conclusion:**

Although the increased number of deaths associated with population aging increased COI, it was expected that the COI would decrease around 2023 due to a decrease in the human capital value of the deceased. In addition, the mortality rate was expected to decrease in the future due to an increase in the percentage of early detection of colorectal cancer via widespread screening and advances in medical technology.

## Introduction

Colorectal cancer (malignant neoplasms of the colon and rectum as per the International Statistical Classification of Diseases and Related Health Problems, Tenth Revision (ICD-10) codes: C18-C20) was the third leading cause of cancer deaths by site worldwide (in both men and women) in 2020 [1]. In Japan, it is the second leading and leading cause of cancer deaths in men (after lung cancer) and women, respectively. Furthermore, both the incidence and mortality rates are increasing, with 52,418 deaths in 2021 (28,080 in men and 24,338 in women). In contrast, in both men and women, the age-adjusted morbidity and mortality rates have remained unchanged and decreased, respectively. This is analogous to the fact that morbidity and the mortality are increasing due to aging [2].

Recently, the effectiveness of colorectal cancer treatment has increased due to highly effective drugs, such as molecular-targeted drugs [3]. Furthermore, early detection has also increased due to the widespread use of fecal occult blood tests and colonoscopies. In Japan, a population-based cancer registry began in the 1950s, and the number of cancer patients nationwide was estimated based on data from several prefectures. The National Cancer Registry System was initiated only in 2016, and data were compiled, analyzed, and managed by the government (the National Cancer Surveys). According to the registry data, colorectal cancer screening uptake rate among people aged 40–69 years increased from 25.8% to 2007 to 44.2% in 2019 [4]. As a result, the percentage of patients with stage 0 or 1 (Union for International Cancer Control Tumor-Node-Metastasis (UICC-TNM) classification) seen before treatment also increased from 43.0% to 2007 to 51.5% in 2019 [5]. Therefore, if colorectal cancer cases are detected at an early stage, morbidity is expected to rise; however, the number of future deaths will decline. Thus, recent trends in colorectal cancer have been changing significantly.

Previous studies attempted to estimate the social burden of colorectal cancer in various countries. Ó Céilleachair et al. conducted a survey on costs of illness studies for colorectal cancer and noted the methodological heterogeneity of the studies and difficulties in comparing them [6]. Similarly, Kriza et al.’s criticisms of cost of illness (hereafter referred to as COI) studies included inconsistencies among different studies, such as different methodologies and cost structures, lack of transparency in reporting, and therefore, concerns regarding their validity [7]. In fact, many recent studies dealt only with direct costs (medical costs), although they used the term “cost of illness” [8–14]. Furthermore, various studies also determined indirect costs, which included those that combined direct costs with the loss of labor and direct costs, loss of labor, and loss of human capital due to death [15–19].

In addition to heterogeneity in definitions, estimates vary widely depending on social conditions and healthcare systems in each country, making it difficult to conduct international comparative studies in absolute terms. Despite these comparative difficulties, COI studies are an excellent way to observe how the burden of one disease compares to that of another in a country and how it changes over time. However, due to limitations in the statistics used to estimate COI, limited comparisons have been made.

Cancer treatment methods have advanced in recent years, and the 5-year survival rate has increased for many cancers. However, recent treatment methods are more expensive than conventional ones, and it is necessary to analyze the cost-effectiveness at the micro level as well as the burden of the disease on society as a whole at the macro level. The cost of illness method [20, 21] as defined by Rice is a method to measure the social burden of disease at the macro level, and by dividing social costs into direct and indirect costs, it makes it possible to provide data for considering efficiency issues at the macro level. It has also influenced policies in many countries. Since colorectal cancer is one of the major cancers among the Japanese population, understanding trends in social costs is considered important.

This study aimed to estimate the social burden of colorectal cancer using the COI method developed by Rice, examine the changes in COI between 1996 and 2020, and predict further changes till 2035. We examined how the recent rapid aging of society affected the social burden and development of medical technology and the widespread use of medical checkups affected early detection.

## Methods

This study used the COI method defined by Rice to examine social burden [20–26]. To examine changes over time, estimates to date (COI estimates from 1996 to 2020) and future projections (COI estimates from 2023 to 2035) were conducted. Estimates were based on hospital admissions, outpatient visits, and deaths in a one-year period via a top-down approach with government statistics. Data used in this study are summarized in Table 1. The government statistics on population and mortality are complete census, whereas the others are sample surveys of the entire population.

**Table 1:** Data sources on the cost of illness (COI)

Source	Issuer	Purpose
Statistics of Medical Care Activities in Public Health Insurance	Ministry of Health, Labour and Welfare	To determine the direct cost of colorectal cancer
Basic Survey on Wage Structure	Ministry of Health, Labour and Welfare	To calculate labor value
Labour Force Survey	Ministry of Internal Affairs and Communications	To calculate labor value
Estimates of Monetary Valuation of Unpaid Work	Cabinet Office	To calculate labor value
Vital Statistics	Ministry of Health, Labour and Welfare	To evaluate the number of deaths
Patient Survey	Ministry of Health, Labour and Welfare	To distinguish the number of patients, total person-days of outpatient visits, and average length of hospital stay
Population Projections for Japan	National Institute of Population and Social Security Research in Japan	To refer future population

Social burden based on the COI method was classified into three categories: direct, morbidity, and mortality costs. Estimation methods for each were as follows. Direct costs included medical expenses for treatment, hospitalization, tests, drugs, and so on, directly caused by the illness. Using the “Statistics of Medical Care Activities in Public Health Insurance,“ the medical expenditure for colorectal cancer was used to calculate annual medical costs. Morbidity cost was the value of labor lost through hospitalization or hospital visits due to illness. For hospitalized patients, it was calculated by multiplying the number of hospitalized patients by sex and the five-year age group, using the “Patient Survey,” and average daily earnings by sex and the five-year age group, calculated by the “Basic Survey on Wage Structure,” “Labor Force Survey,” and “Estimation of the Monetary Value of Unpaid Labor.” Similarly, morbidity costs for outpatients were calculated by multiplying the number of outpatients by sex and the five-year age group by 1/2 of the average daily earnings by sex and the five-year age group, followed by summing the results. This was based on the assumption that one day and half a day of work time was lost in the case of a hospitalization and hospital visit, respectively. Since the 1996 data is based on the estimated number of outpatients and inpatients in the 10-year age groups, the ratio per population was calculated in the 10-year age groups and multiplied by the 1996 population in the 5-year age group.

Mortality cost, as a social cost of human capital loss, was considered as the total amount of income that the deceased person would have earned in the future if they had not died. First, the number of deaths due to colorectal cancer was obtained by sex and five-year age group. Assuming that the deceased survived from the age at death to average life expectancy, the total income was calculated as the present value at a discount rate of 3% via the “Basic Survey on Wage Structure,“ “Labor Force Survey,“ and “Estimation of Monetary Values of Free Labor.“ In addition, as a sensitivity analysis, we varied the discount rate from 0 to 5% and observed the impact.

In the Japanese healthcare system, almost all medical costs were covered and reimbursed by the public medical insurance, except for additional services not directly related to medical care, such as private room charges. “Statistics of Medical Care Activities in Public Health Insurance” was likely to reflect the costs for medical services. Social burden caused by long-term care was not considered. This was since the cost of long-term care for malignant neoplasms was considered to be as low as approximately 3% of the total cost [27] and had little impact on the overall COI.

For future projections of the societal burden of colorectal cancer, projections that used models with Markov chains have been reported [28–30]. However, in such models, it was difficult to obtain the transition probabilities between stages necessary for estimation from statistical data. Furthermore, most studies used transition probabilities obtained using the calibration performed by Tappenden et al. [31], which did not consider subsequent changes in medical technology. Therefore, in this study, we obtained statistical data on the annual number of hospitalizations and outpatient visits per population, average length of stay, and mortality rates by sex and five-year age group. Furthermore, we used every three-year time point from 1996 to 2020 to create approximate estimates using exponential or log approximation if the variables were in a decreasing or increasing trend, respectively. Future estimates of direct costs were obtained by dividing historical direct costs into inpatient and outpatient costs, dividing each by the total number of inpatient days and outpatient visits to obtain unit costs, estimating the trend using an exponential/logarithmic approximation curve, and multiplying by the estimated total number of inpatient days and outpatient visits to obtain a total sum. Details of the actual COI calculation methodology are detailed in Matsumoto (2022) [32]. In addition, the impact of the recent spread of colorectal cancer screening was considered using the available incidence data by stage according to UICC-TNM classification from 2008 to 2019. Trends in incidence rates were determined and aggregated to produce future projections on the number of colorectal cancer cases by stage using the same estimation method as for the COI future projections by sex and five-year age group. Specifically, “Patient surveys” were used to obtain the estimated incidence of colorectal cancer by sex and age group from 2008 to 2017, based on the National Cancer Center’s Annual Report of Hospital-Based Cancer Registries, and the number of patients in each stage, excluding unknowns, was calculated. The number of patients per stage was obtained by multiplying the proportion of patients in each stage by the total number of patients, assuming no difference in age. This was used to calculate trends in prevalence rates and to make projections for the future.

## Results

Table 2 shows the trends in COI and related indicators. In 2020, the COI was 1,451.0 billion yen (approximately 10.7 billion USD). The breakdown was 417.3, 74.2, and 959.5 billion yen for direct, morbidity, and mortality costs, respectively. The COI gradually increased since 1996, with a 1.71-fold increase over the 24-year period from 1996 to 2020. Regarding annual percent change (APC), this represented an increase of 2.4%. Mortality costs, which accounted for more than 60% of the total COI, have continued to increase. The contribution rate of direct costs was 42.0%, and mortality and direct costs together explained 96.3% of the increase in the COI from 1996 to 2020.

**Table 2:** The time trend of cost of illness (COI) of colorectal cancer

	1996	1999	2002	2005	2008	2011		2014	2017	2020
Population (thousand person)	125,864	126,686	127,435	127,768	127,692	127,799		126,949	126,707	123,214
[% of those 65 years or older]	15.1%	16.7%	18.5%	20.2%	22.1%	23.3%		26.1%	27.7%	28.7%
Number of deaths (person)	32,617	35,360	37,668	40,830	43,006	45,563		48,481	50,679	51,785
[% of those 65 years or older]	69.4%	72.5%	75.3%	77.2%	79.6%	80.2%		82.6%	85.5%	87.2%
Number of incidence (person)	83,108	90,289	105,195	104,056	112,772	124,921		135,434	153,189	155,625※
[% of those 65 years or older]	60.8%	64.0%	65.9%	67.7%	70.9%	70.4%		73.5%	76.4%	77.2%
Crude mortality/incidence rate	39.2%	39.2%	35.8%	39.2%	38.1%	36.5%		35.8%	33.1%	33.3%
Average age of incidence (year)	67.2	68.1	68.7	69.4	70.4	70.7		71.0	71.6	71.9
Average age of death (year)	70.3	71.2	72.2	73.1	74.0	74.6		75.1	75.9	76.5
Direct cost (billion yen)	165.2	250.8	289.4	274.9	367.8	367.5		436.8	460.1	417.3
Morbidity cost (billion yen)	51.8	61.7	63.5	71.4	64.0	66.1		67.8	68.9	74.2
Mortality cost (billion yen)	633.3	630.2	729.9	723.9	728.4	821.4		837.2	897.9	959.5
COI (billion yen)	850.3	942.7	1,082.8	1,070.2	1,160.2	1,255.0		1,341.8	1,427.0	1,451.0
COI per person (yen)	6,755.5	7,440.9	8,497.0	8,376.3	9,085.6	9,819.9		10,569.5	11,262.2	11,776.2

Source of population: Ministry of Internal Affairs and Communications “Population Estimates”

Source of the number of cancer deaths: “Vital Statistics”

Source of the number of incidence: Center for Cancer Control and Information Services, National Cancer Center, Japan.

Average age of incidence: Calculated according to the number of incidence.

Average age of death: Calculated according to the number of deaths, sex and age (five-year old age-grade), cause of death in “Vital Statistics.”

※ 2019 data.

Colorectal cancer is a disease with higher incidence and mortality rates in the older adult age group. The incidence rate rose around 40 years. In 2020, the incidence rate per 100,000 persons was 26.7 for men and 22.7 for women in the 40–44 years age group. However, it was 508.6 for men and 365.2 for women in the 85 + years age group (19.1 and 16.1 times higher, respectively). The mortality rate per 100,000 was 3.9 for males and 3.5 for females in the 40–44 years age group. However, it was 322.6 for males and 249.8 for females in the 85 + years age group (82.4 times and 71.6 times higher, respectively). Hence, an increase in the older adult population due to societal aging had a major impact on changes in the COI.

The increase in mortality and direct costs was largely due to an increase in the number of deaths: between 1996 and 2020, the number of deaths increased by a factor of 1.59 (APC: 2.0%). Although the case fatality rate declined from 39.2% to 1996 to 33.3% in 2020, the effects of the rapid aging of society outweighed the impact of the decline in fatality rates, which caused the number of deaths to rise. Effects of social aging also caused an increase in the average age of morbidity and mortality, and the average ages of morbidity and mortality increased by 4.73 years and 6.21 years from 1996 to 2020, respectively.

Future estimates of the COI using historical trends in health-related indicators (mortality rate, number of outpatient visits and hospitalizations per population, and average length of hospital stay) indicated a decrease of 6.4% from 1,451.0 billion yen in 2020 to 1,357.8 billion yen in 2035 (Table 3). The breakdown showed that while direct costs would increase by 8.9%, morbidity costs and mortality costs would decrease by 23.8% and 11.7%, respectively. The contribution of each of these costs to the decrease in COI was − 39.6%, 18.9%, and 120.7%, respectively. The number of deaths continued to rise till 2035 (APC was 0.69%), while the average age at death continued to rise, and increased by 2.60 years from 2020 to 2035. The number of incidence also rose (APC was 0.65%). However, the average age at incidence increased by 1.93 years. In addition, the case fatality rate was expected to remain roughly flat during this period. Table 4 shows the estimates of the COI by varying the discount rate from 0 to 5%. However, the COI trend was not significantly affected.

**Table 3:** Future prediction of cost of illness (COI) of colorectal cancer (2% discount rate)

Item	2020	2023	2026	2029	2032		2035
Estimated population (thousand person)	123,214	123,751	121,903	119,850	117,616		115,216
[% of 65 years or older]	28.7%	29.6%	30.2%	30.9%	31.6%		32.8%
Number of deaths (person)	51,785	54,165	55,031	56,354	56,504		57,446
[% of 65 years or older]	87.2%	87.5%	87.9%	88.3%	88.6%		89.5%
Number of incidence (person)	148,544※	153,823	157,509	160,714	162,317		163,796
[% of 65 years or older]	76.6%	77.1%	77.1%	77.1%	77.2%		78.1%
Crude mortality/incidence rate	34.9%	35.2%	34.9%	35.1%	34.8%		35.1%
Average age of incidence (year)	72.3	72.9	73.2	73.7	73.9		74.2
Average age of death (year)	76.5	77.2	77.7	78.3	78.6		79.1
Direct cost (billion yen)	417.3	459.8	463.0	464.6	460.7		454.2
Morbidity cost (billion yen)	74.2	71.3	67.6	64.0	60.4		56.5
Mortality cost (billion yen)	959.5	947.3	923.2	898.8	872.6		847.0
COI (billion yen)	1,451.0	1,478.4	1,453.9	1,427.3	1,393.7		1,357.8
COI per person (yen)	11,776.2	11,946.8	11,926.4	11,908.8	11,849.5		11,784.9

Source of population: Ministry of Internal Affairs and Communications “Population Estimates”

Source of the number of cancer deaths: “Vital Statistics”

Source of the number of incidence: Center for Cancer Control and Information Services, National Cancer Center, Japan.

Average age of incidence: Calculated according to the number of incidence.

Average age of death: Calculated according to the number of deaths, sex and age (5 years old age-grade), cause of death in “Vital Statistics”.

2020 values are actual values (* is 2019 value)

**Table 4:** Sensitivity analysis of COI by discount rate

billion yen							
		0%	1%	2%	3%	4%	5%
1996		1,022.6	955.7	898.9	850.3	808.2	771.5
1999		1,109.5	1,044.9	989.9	942.7	901.7	866.0
2002		1,282.1	1,204.6	1,139.0	1,082.8	1,034.3	992.0
2005		1,264.0	1,188.8	1,125.0	1,070.2	1,022.8	981.5
2008		1,350.4	1,276.8	1,214.1	1,160.2	1,113.4	1,072.5
2011		1,471.4	1,387.6	1,316.3	1,255.0	1,201.8	1,155.4
2014		1,563.4	1,477.5	1,404.5	1,341.8	1,287.5	1,240.1
2017		1,666.6	1,573.6	1,494.7	1,427.0	1,368.4	1,317.3
2020		1,697.9	1,602.3	1,520.9	1,451.0	1,390.3	1,337.3
2023		1,718.0	1,625.4	1,546.4	1,478.4	1,419.4	1,367.7
2026		1,684.0	1,595.1	1,519.2	1,453.9	1,397.0	1,347.2
2029		1,647.9	1,562.8	1,490.0	1,427.3	1,372.6	1,324.7
2032		1,605.1	1,523.6	1,453.9	1,393.7	1,341.2	1,295.1
2035		1,559.8	1,482.0	1,415.4	1,357.8	1,307.5	1,263.3

**Fig. 1:**
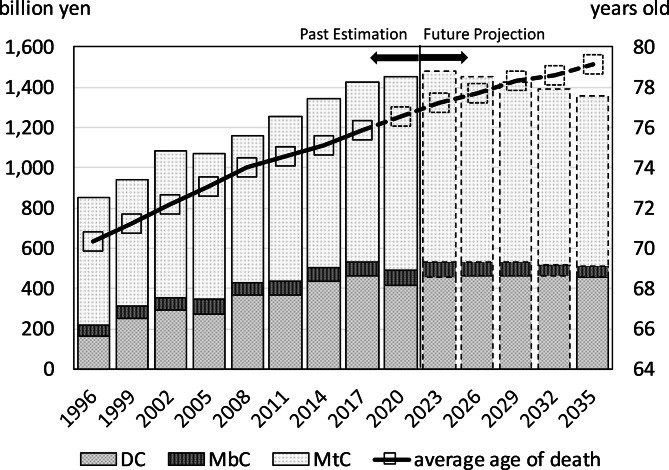
Cost of illness (COI) projection with cost element and the average age at death

Past COI estimates and future projections till 2035 are illustrated in Fig. 1. COI peaked in 2023 and subsequently began to decline. Direct costs continued to increase till 2017. However, they subsequently leveled off. Furthermore, mortality costs were expected to decline by approximately 2023. During this period, the average age at death continued to rise.

**Fig. 2:**
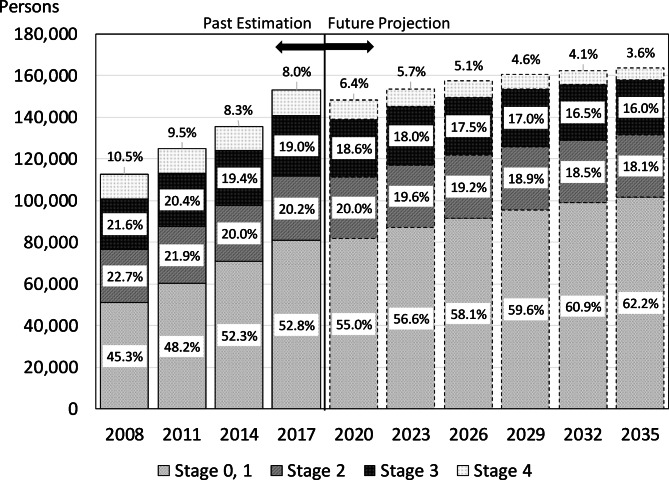
The number of colorectal cancer incidence by stage and its projection Data were estimated using “Annual Report of Hospital-Based Cancer Registries” and “Patient Survey.“

Figure 2 shows the number of colorectal cancer incidence by stage and its projection. Since the incidence data were only available up to 2019, future estimates will be made from 2020. However, the number of cases was expected to continue to increase in the future. Nevertheless, by stage, we saw that the proportion of patients detected at stage 0, 1 rose, while the proportion of patients detected at higher stages declined.

## Discussion

Estimations of the COI from 1996 to 2035 showed that the COI for colorectal cancer would peak in 2023 and subsequently decline in the future. This was largely due to changes in mortality costs, which accounted for 60–70% of the COI. Direct costs were expected to rise until 2036, while morbidity costs were expected to account for a smaller percentage of the total, approximately 5–6%. In past estimates, the contribution rate of direct costs was as high as 42.0% due to the upward trend in COI. However, in future estimates, the contribution rate of direct costs was negative due to the downward trend in the COI. Conversely, the contribution rate of mortality costs was 54.3% in the past estimates. However, it was 120.7% in the future estimates, which suggested that the COI changed in a way that was pulled along by the change in mortality costs from an upward to a downward trend.

Change in mortality costs was thought to be largely due to the aging of society. Societal aging increased the overall number of deaths from colorectal cancer, which was characterized by high mortality rates in the older age groups. Since mortality rates in each age group consistently declined, the overall increase in deaths could be attributed to population aging. However, population aging also increased the average age at death for colorectal cancer, which resulted in a decrease in the per capita human capital value. Decline in mortality costs was expected to occur between 2023 and 2026 as the impact of the decline in human capital value outweighed the impact of the increase in the number of deaths.

During this period, the number of incidence consistently rose in past estimates and was expected to continue to rise until 2035 in future estimates. By age group, the mortality rate declined in all the age groups, except for females aged 85 and older. Meanwhile, the incidence rate remained unchanged or slightly increased in all the age groups. Thus, the fatality rate, which was the mortality rate divided by the incidence rate, was expected to decline slightly until 2020 and remain unchanged after 2020. This may be due to the fact that when mortality and incidence rates were compared by sex and age group, the difference between the younger and older age groups was larger for the mortality rate than incidence rate. The incidence rate per 100,000 persons was 19.1 times higher for males and 16.1 times higher for females in the 40–44 years age group versus the 85 + age group. However, the mortality rate was 82.4 times higher for males and 71.6 times higher for females. The effect of the aging of the population was more apparent in the number of deaths than number of incidence.

Societal aging has a greater impact on the increase in mortality than incidence. However, the reason why the fatality rate has not increased significantly may be due to the widespread use of colorectal cancer screening, in addition to advances in medical technology. As shown in Fig. 2, although the number of incidence is rising, the number of patients detected in stage 3 and 4 is expected to decline. Furthermore, the proportion of patients detected in stage 1 is expected to increase significantly from 2008 to 2035. In fact, according to estimates using “Comprehensive survey of Living Condition of the people on health and welfare,” during the 10-year period from 2010 to 2019, the colorectal cancer screening uptake rate for those aged 40 to 69 years increased from 28.1 to 47.8% for men and from 23.9 to 40.9% for women [33]. This was thought to be linked to the early detection of colorectal cancer. If colorectal cancer screening uptake rate further increased in the future, early detection is expected to increase, which may lead to a decrease in the mortality rate. The impact of the advances in medical technology has also been significant. Development of endoscopic surgery and advances in chemotherapy, such as molecular-targeted drugs, have also increased the five-year survival rate. According to data from the National Council of Cancer Centers, the five-year survival rates for patients with stage 3 and stage 4 diagnosed in 2001–2003 were 76.2% and 15.0%, respectively, and for patients with stage 3 and stage 4 patients diagnosed in 2011–2013 had increased five-year survival rates of 85.8% and 23.3%, respectively [34].

The rank order of incidence of cancer by site in Japan is colon, lung, stomach, breast, and prostate from first to fifth, and the rank order of death is lung, colon, stomach, pancreas, and liver, in that order. The authors have estimated the COI of these cancers, excluding pancreatic cancer [32, 35, 36]. Among these cancers, stomach and liver cancers have already shown a decrease in COI and are expected to decrease further in the future. Lung and colorectal cancers have shown an increasing trend to date, but are expected to decrease in the future. Breast and prostate cancers are expected to continue to increase. Colorectal and lung cancers show similar trends, and moreover, the same is true for the future, as lower mortality costs will lead to lower overall COI. In the near future, the importance of the societal burden of site-specific cancers may be reversed.

This study has several limitations. First, our regression curves should be interpreted with caution. Our projections were based on data collected over a relatively short period, during which the healthcare system in Japan experienced major restructuring (e.g., introduction of a case-mix reimbursement system and functional differentiation between acute and non-acute beds). However, to create estimates for the relatively near future, projections based on past trends were considered more appropriate than estimates based on models that involved difficulties in determining transition probabilities. Second, with regard to future projections, wage levels were fixed to those in 2020. This reflects the extremely low rate of wage growth in Japan in recent years, but may be an underestimate. Third, the use of the COI method obscures whose costs were being estimated. However, there were many stakeholders involved in medical care, and from the perspective of each stakeholder, such as patients, medical institutions, governments, and taxpayers, it was conceivable that various changes in disease could alter the costs for each stakeholder in a different direction. Rather, comprehensive cost estimation from the standpoint of social cost would contribute to the prioritization of policies for the disease.

## Conclusion

The number of deaths and incidence from colorectal cancer has been and will continue to rise due to the aging of society. However, the social cost of colorectal cancer is expected to peak by approximately 2023 and subsequently decline. In addition, the fatality rate of colorectal cancer is expected to decrease in the future due to an increase in the percentage of patients detected at an early stage by an increase in screening and the development of medical technologies, such as endoscopic surgery and chemotherapy.

## Data Availability

All data used in the analysis are public data and can be obtained from the following website: https://www.e-stat.go.jp/.
